# A Diversity of Cell Types, Subtypes and Phenotypes in the Central Nervous System: The Importance of Studying Their Complex Relationships

**DOI:** 10.3389/fncel.2020.628347

**Published:** 2020-12-23

**Authors:** Marie-Ève Tremblay

**Affiliations:** ^1^Axe Neurosciences, Centre de recherche du CHU de Québec - Université Laval, Québec, QC, Canada; ^2^Département de Médecine Moléculaire, Université Laval, Québec, QC, Canada; ^3^Department of Neurology and Neurosurgery, McGill University, Montréal, QC, Canada; ^4^Division of Medical Sciences, University of Victoria, Victoria, BC, Canada; ^5^Department of Biochemistry and Molecular Biology, Faculty of Medicine, The University of British Colombia, Vancouver, BC, Canada

**Keywords:** microglia, astrocyte, oligodendrocyte lineage cell, peripheral immune cell, subtypes, diversity, interactions, CNS

All the cell types in the central nervous system (CNS) cooperate to mediate proper development, function, and plasticity. Similarly, brain repair and neuroprotection, but also demyelination, synaptic loss and neurodegeneration, were increasingly shown to involve non-neuronal cells—both glial cells and peripheral immune cells—among the CNS parenchyma. Adding another degree of complexity, the non-neuronal cell populations are emerging as comprised of different subtypes, endowed with unique properties and functions at steady-state, and which can adopt various phenotypes upon exposure to homeostatic challenges. As a consequence, studying the multidirectional relationships between these different cell types, subtypes and phenotypes in the CNS is now required to provide insights into the mechanisms underlying physiological processes such as neuronogenesis, axon guidance, myelination, vascular formation and remodeling, regulation of neuronal activity, as well as synaptic formation, function and plasticity, and behavioral outputs, among other essential CNS functions.

## A Diversity of Cell Types, Subtypes, and Phenotypes

In the CNS, the different types of neurons, which are identified by their neurotransmitters, neurotrophic or neuroprotective factors (Que et al., 2019; Sugino et al., 2019; Cizeron et al., 2020), are accompanied by non-neuronal cells showing similar, or greater levels of heterogeneity. This cellular diversity pertains to glial cells (microglia, astrocytes, cells of the oligodendrocytic lineage) and the peripheral immune cells that can infiltrate the CNS (Oberheim et al., 2012; Butt and Verkhratsky, 2018; Foerster et al., 2019; Stratoulias et al., 2019; Dumas and Prinz, 2020). While the microglial field rejected the M1 and M2 categorization (Martinez and Gordon, 2014; Ransohoff, 2016), the A1 and A1 astrocytic dichotomy is increasingly controversial (Oberheim et al., 2012; Verkhratsky and Nedergaard, 2018; Khakh and Deneen, 2019; Westergard and Rothstein, 2020). Cells of the oligodendrocytic lineage are also emerging as a diverse population with different subsets co-existing among the CNS (Foerster et al., 2019). Similarly, peripheral immune cells comprised of granulocytes (neutrophils, eosinophils, and basophils), monocytes, and lymphocytes (T cells and B cells) are highly heterogeneous, and accompanied by additional CNS border-associated populations, yet the nature of the cells infiltrating the CNS, and their distinctive properties once in the CNS environment are less understood (Theret et al., 2019; Dumas and Prinz, 2020). Accumulating findings reveal that glial and peripheral immune cells display distinct subsets that vary in their localization, epigenetic signature, protein and gene expression, but also in their morphological and ultrastructural attributes (Bisht et al., 2016; Ayata et al., 2018; Silvin and Ginhoux, 2018; Foerster et al., 2019; Khakh and Deneen, 2019; Stratoulias et al., 2019; Burns et al., 2020; Castellani and Schwartz, 2020; St-Pierre et al., 2020; Tan et al., 2020; Westergard and Rothstein, 2020). Whether the non-neuronal subsets in the CNS indicate the existence of different subtypes, with unique intrinsic properties and specialized functions, or reflect phenotypes that change their properties and functions upon exposure to challenges, or both, remains in most cases elusive. Understanding how the glial and peripheral immune cell heterogeneity determines varied functions in the CNS, at steady-state and upon various challenges, is importantly required to design cellular interventions that specifically target (modulate, stimulate or inhibit) microglia, astrocytes, oligodendrocytic lineage cells or peripheral immune cells performing contextually-desirable or undesirable functions.

## The Challenge of Studying Their Complex Relationships

Distinguishing between glial and immune cell subtypes and phenotypes in the CNS requires fate mapping tools that allow to follow individual cells over time. Such approaches, which are becoming increasingly available, have been used by pioneering investigations to determine the origin of tissue resident macrophages, including microglia (Prinz et al., 2014; Perdiguero and Geissmann, 2016), or the turnover and longevity of microglia within the CNS (Askew et al., 2017; Füger et al., 2017; Tay et al., 2017b), among other important findings. To complement the fate mapping strategies, *in situ* and *in vivo* approaches should be prioritized to study the biological relevance of glial and immune diversity in the CNS, considering that *in vitro* and *ex vivo* preparations modify non-neuronal cell responses (Hellwig et al., 2013; Gosselin et al., 2014). Recent technological advances such as single cell transcriptome analysis have revealed an unprecedented heterogeneity in the non-neuronal cells of the CNS's molecular signatures (Krasemann et al., 2017; Hammond et al., 2019; Kierdorf et al., 2019; Deczkowska et al., 2020). Nevertheless, whether the glial and immune cells in the CNS are multitasking or perform specialized functions still remains largely undetermined. Considering that essential CNS functions emerge from the dynamic interactions between all cell types in the CNS, including learning and memory, judgement, emotional transformation, decision making, as well as behavioral outputs, it would be essential to unravel the complex relationships between cell types, subtypes, and phenotypes of neuronal and non-neuronal cells in the CNS. The neurons, microglia, astrocytes, and oligodendrocytic lineage cells have been shown to interact with another, structurally and functionally, and they communicate with the peripheral leukocytes infiltrating the CNS or transiting via the perivascular space and other CNS borders (Neuroimmune Communication, 2017; Tay et al., 2017c; Carrier et al., 2020). Microglia can control astrocytic functions, and vice versa, astrocytes can influence microglia, and the same holds for the reciprocal crosstalks taking place between astrocytes, microglia, and oligodendrocytic lineage cells (Béchade et al., 2013; Kettenmann et al., 2013; Domingues et al., 2016; Liddelow et al., 2017; Matejuk and Ransohoff, 2020). Within this integrative viewpoint, collaborative research endeavors that bridge complementary expertise with the different non-neuronal cell types, subtypes and phenotypes, are now required to provide maximal insights into the functional relevance of these multidimensional relationships within the CNS.

## The Individual Circumstances Increasing Variability

Non-neuronal cell types, subtypes and phenotypes vary between CNS regions, stages of life, sex, specie, and context of health or disease (Silvin and Ginhoux, 2018; Foerster et al., 2019; Khakh and Deneen, 2019; Stratoulias et al., 2019; Castellani and Schwartz, 2020; Tan et al., 2020; Westergard and Rothstein, 2020). Their diversity is modulated by the genetic vulnerabilities, environmental challenges (e.g., stress, infection, pollution), lifestyle factors (e.g., diet, sleep, physical activity, alcohol, cannabis), peripheral comorbidities (e.g., asthma, colitis, arthritis) and other individual circumstances (e.g., exposure to nature, social support), thus tremendously increasing the variability (Hanamsagar and Bilbo, 2017; Tay et al., 2017a; Savage and Tremblay, 2019; Madore et al., 2020). The non-neuronal cells further display differences in their CNS colonization, maturation, gene and protein expression, morphology, ultrastructure, function, and response to challenges between the sexes (Schwarz and Bilbo, 2012; Hui St.-Pierre et al., 2018; Nelson et al., 2019; Bordeleau et al., 2020; Yasuda et al., 2020). In the periphery, the concept of “immunobiography” has been formulated, to reflect the tremendous inter-individual differences in immune function cumulating during life, and propose the idea that the individual immune signature can inform in a very sensitive manner on the state of health or disease, and allow to predict the outcome of various treatment strategies (Del Giudice et al., 2017; Franceschi et al., 2017). Similarly, the physiological and immune functions of both microglia and astrocytes are regulated by a multitude of external influences, which can induce cellular memory and epigenetic remodeling (Reemst et al., 2016; Valero et al., 2016; Tay et al., 2017a; Ayata et al., 2018; Murphy-Royal et al., 2019; Wheeler et al., 2019; Madore et al., 2020). As a consequence, elucidating how various individual circumstances guide the multidimensional relationships between neuronal and non-neuronal cell types, subtypes and phenotypes in the CNS, is expected to provide unprecedented opportunities to develop personalized treatment strategies for a wide variety of neurodevelopmental, neuropsychiatric and neurodegenerative diseases in which the neuronal and non-neuronal cells of the CNS are together critically involved.

## Author Contributions

The author confirms being the sole contributor of this work and has approved it for publication.

## Conflict of Interest

The author declares that the research was conducted in the absence of any commercial or financial relationships that could be construed as a potential conflict of interest.
